# Fewer Reoperations After Lumpectomy for Breast Cancer with Neoadjuvant Rather than Adjuvant Chemotherapy: A Report from the National Cancer Database

**DOI:** 10.1245/s10434-016-5760-8

**Published:** 2017-01-06

**Authors:** Jeffrey Landercasper, Barbara Bennie, Benjamin M. Parsons, Leah L. Dietrich, Caprice C. Greenberg, Lee G. Wilke, Jared H. Linebarger

**Affiliations:** 1 Department of Medical Research, Gundersen Medical Foundation, La Crosse, WI USA; 2 Norma J. Vinger Center for Breast Care, Gundersen Health System, La Crosse, WI USA; 3 Department of Mathematics and Statistics, University of Wisconsin La Crosse, La Crosse, WI USA; 4 Department of Medical Oncology, Gundersen Health System, La Crosse, WI USA; 5 Department of Surgery, University of Wisconsin Madison, 600 Highland Avenue, Madison, WI USA; 6 Department of Surgery, Gundersen Health System, La Crosse, WI USA

## Abstract

**Background:**

Reoperations occur frequently after initial lumpectomy for breast cancer. The authors hypothesized that the receipt of neoadjuvant chemotherapy (NAC) is associated with fewer reoperations.

**Methods:**

The association between timing of chemotherapy and reoperation rates (ROR) after lumpectomy was investigated for patients with stages 1–3 breast cancer in the National Cancer Database (NCDB) from 2010 to 2013 by multivariable logistic regression modeling. Then propensity score-matching was performed.

**Results:**

The unadjusted ROR for 71,627 stages 1–3 patients was 11.4% for those who had NAC compared with 20.3% for those who had postoperative chemotherapy (*p* < 0.001) (odds ratio [OR] 0.53; 95% confidence interval [CI] 0.49–0.57; *p* < 0.001). The ORs for the reoperations performed for patients with stages 1, 2, and 3 cancers who received NAC were respectively 0.65 (95% CI 0.56–0.75), 0.50 (95% CI 0.45–0.56), and 0.27 (95% CI 0.19–0.38) The *p* values for all were lower than 0.001.

**Conclusion:**

For a population of patients receiving chemotherapy, the receipt of chemotherapy before instead of after surgery was associated with fewer reoperations after initial lumpectomy for breast cancer.

More than one in five patients treated with initial lumpectomy for breast cancer need another operation for perceived residual disease.^1^–^4^ In addition, there is strong evidence for marked variability of reoperation rates (RORs) among both individual surgeons and individual institutions.^1^–^3^ Consequently, exploration of new methods of care is needed to limit reoperations.^5^ This study aimed to investigate whether the timing of chemotherapy is associated with a lower ROR in patient populations for whom this treatment is appropriate.

Neoadjuvant chemotherapy (NAC) reduces the cancer burden before operation for many patients, offering the patient a better chance of successful breast conservation rather than mastectomy.^6^ The number of patients with newly diagnosed breast cancer who receive NAC is increasing, but it still comprised less than 17% of all patients undergoing operations recently reported in the National Cancer Database (NCDB).^6^ For a population of patients receiving chemotherapy, we hypothesized that receipt of NAC is associated with fewer reoperations after lumpectomy. We used the NCDB to test this hypothesis.

## Methods

The NCDB contains de-identified, Health Insurance Portability and Accountability Act (HIPAA)-compliant participant user files. All patient identifiers are removed. Thus, institutional review board approval is not required. “The NCDB is a joint project of the American College of Surgeons Commission on Cancer and the American Cancer Society. The hospitals participating in the NCDB are the source of the de-identified data used herein; they have not verified and are not responsible for the statistical validity of the data analysis or the conclusions derived by the authors.”^7^,^8^

### Study Population

The inclusion criteria specified female patients older than 18 years receiving chemotherapy for clinical stages 1–3 invasive breast cancer who underwent initial lumpectomy during the years 2010 to 2013. Patients with non-primary breast cancer histologic codes, diagnosis by excisional rather than needle biopsy, missing values for reoperations, missing days from diagnosis to the first surgical procedure or a definitive surgical procedure, or missing predictor variables (chemotherapy timing) were excluded from the study. Patients coded as having 0 days from diagnosis to the first surgical procedure also were excluded. Patients who had missing values for confounding variables were treated as a separate category (unknown). A patient cohort identical to the aforementioned patients except for age restricted to older than 70 years also was used in separate modeling.

### Primary Outcome

The primary outcome variable was ROR within 60 days after the initial lumpectomy for invasive breast cancer. A reoperation could be either a lumpectomy or a mastectomy. Reoperation rate is not an NCDB data field. A reoperation can be identified by the fields of “first surgical procedure, days from diagnosis” and “definitive surgical procedure, days from diagnosis.” If the latter is greater than the former, then a reoperation occurred.^2^ Patients with 0 days between first and definitive procedures were excluded from the study. Thus, patients with excisional biopsy for first surgery and patients with needle biopsy and definitive surgery on the same day also were excluded.

### Independent Variables

**Fig. 1:**
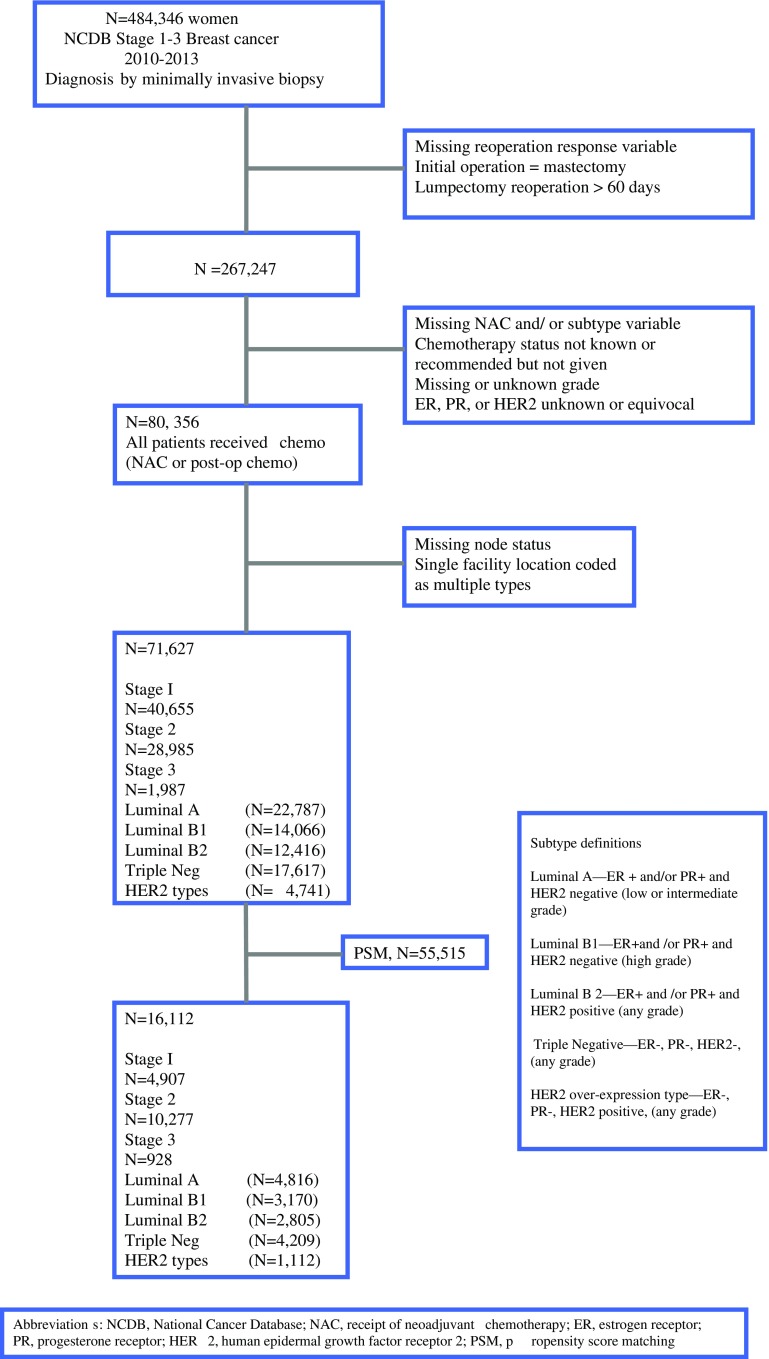
CONSORT diagram

The primary predictor variable was receipt of NAC, determined by comparing days to initial surgery and days to initial chemotherapy. The ROR for the patients with receipt of NAC was compared with that for the patients receiving postoperative chemotherapy. A secondary predictor variable was breast cancer subtype. Because information on breast cancer subtypes based on multigene signature testing is limited to a small proportion of all breast cancer patients in the NCDB, we used the St. Gallen immunohistochemistry (IHC) surrogate subtypes^9^–^15^ (Fig. 1). Patients with a borderline values for estrogen receptor (ER), progesterone receptor (PR), or human epidermal growth factor 2 (HER2) were excluded from the study.

In the multivariable modeling, the independent variables included patient, facility, and tumor variables previously used by NCDB investigators for ROR, as well as those used in investigations from other databases.^1^–^4^,^16^ These variables included patient age, race, insurance status, comorbidities (Charlson/Deyo Score), education, income, tumor size, node status, and facility type, location, and volume. When a single facility type was coded as more than one type or unknown, we created “multiple” and “unknown” categories. When phenotypic IHC cancer subtypes were used as a predictor variable, then hormone receptor status, HER2 status, and tumor grade were excluded as covariates in the regression models.

### Statistical Analysis

The Cochrane Armitage test was used to determine trends in ROR from 2010 to 2013. A univariate (unadjusted) computation of NAC, breast cancer subtypes, and all covariates with ROR was performed using *χ* ^2^ tests. Multiple multivariable logistic regression analyses were performed to characterize the association between receipt of NAC, cancer subtypes, and ROR, with adjustment for all the confounding variables. For all tests, *p* values lower than 0.05 were considered significant. The models were first run for all patients older than 18 years, then repeated for patients older than 70 years.

A second set of models, including all patients receiving chemotherapy, comprised matched cases (those receiving NAC) and control subjects (those not receiving NAC) according to propensity scores. The propensity scores were estimated probabilities of patients receiving NAC based on patient age and tumor characteristics (size, nodal status, and clinical stage). A boundary was set requiring that the propensity score of two patients (one receiving NAC and one not receiving NAC) must differ by less than 0.10 when cases and control subjects were matched. A logistic model with matched pairs then was used to model the likelihood of reoperation based on NAC, with adjustment for confounding variables related to patient and facility characteristics. The CONSORT diagram for the models is seen in Fig. 1.

Next, models were developed to determine whether an association existed between breast cancer subtypes and ROR in a patient cohort receiving NAC. After identification of a significant association, a model was developed to determine whether the association between subtypes and ROR persisted when the cohort was restricted to include only the patients with a pathologic complete response (pCR) to NAC. All statistical analyses were performed with SAS software, version 9.3 (SAS Institute Inc., Cary NC, USA).

## Results

The primary analysis comprised 71,627 initial lumpectomy patients. All received chemotherapy either pre- or postoperatively. Overall, the mean and median (range) of elapsed days from diagnosis to first surgical procedure were respectively 53 and 30 days (range 1–1241 days). In the reoperation group, these periods were respectively 23 and 21 days (range 1–60 days). After exclusion of cases with missing values for these fields, the number of days from diagnosis to first surgical procedure and to definitive surgical procedure were equal among all the patients who did not undergo reoperation. The mastectomy rate was 4.2% (510/12,157) in the NAC group and 7.6% (4521/59,470) in postoperative adjuvant group (*p* < 0.001).

**Fig. 2:**
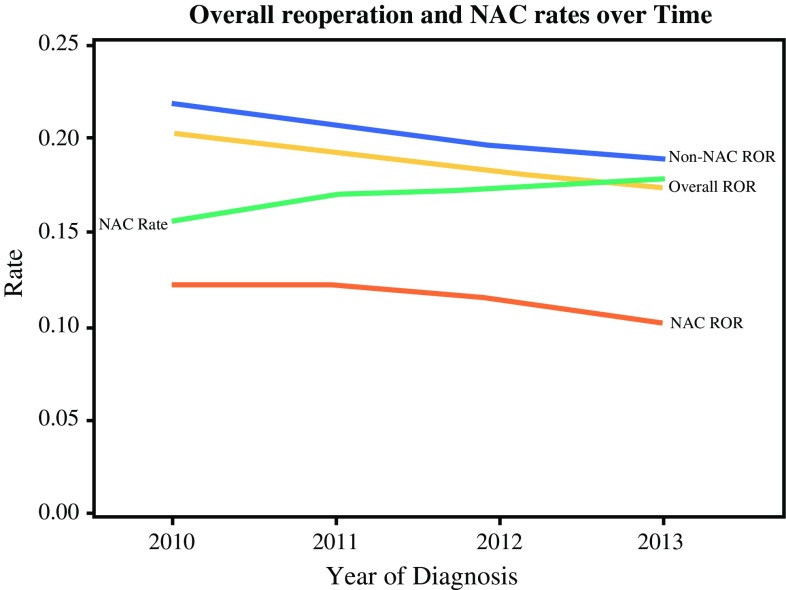
Trends for reoperation rates and receipt of neoadjuvant chemotherapy. *ROR* reoperation rates after lumpectomy for invasive breast cancer, *NAC* receipt of neoadjuvant chemotherapy, *NAC rate* proportion of Stages 1–3 breast cancer patients receiving NAC

The patients with and without receipt of NAC were compared. The proportion of patients receiving NAC was 17%, increasing from 15.6% in 2010 to 17.9% in 2013 (*p* < 0.001). Concurrently, the ROR for all the patients in this group was 18.8%, decreasing from 20.4% in 2010 to 17.4% in 2013 (*p* < 0.001) (Fig. 2). Of all reoperations, 8413 (62.6%) were the lumpectomy type and 5031 (37.4%) were the mastectomy type.

**Fig. 3:**
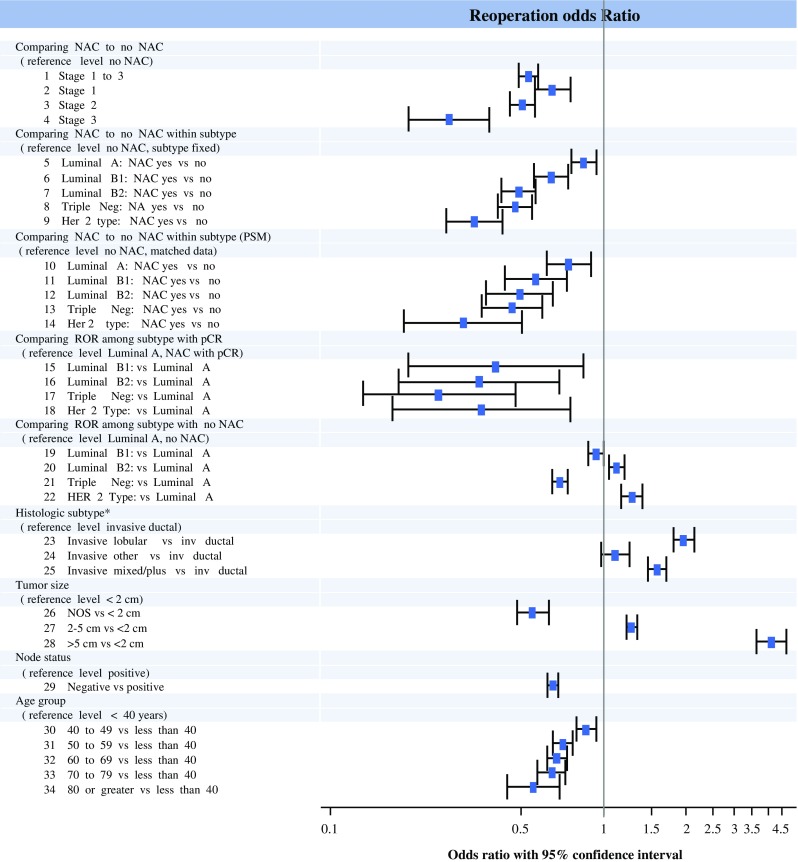
Forest plot of associations between neoadjuvant chemotherapy receipt and lumpectomy reoperation rates in Stages 1–3 breast cancer. *Asterisk* Histologic categories: Invasive ductal, invasive lobular, invasive “mixed/plus” (invasive ductal plus other invasive type and/or DCIS; excludes LCIS), invasive other (all others not previously specified). *NAC* receipt of neoadjuvant chemotherapy, *PSM* propensity score matching, *pCR* pathologic complete response to neoadjuvant chemotherapy

A significant association was observed between the use of NAC, breast cancer subtypes, and ROR when control was used for all the confounding variables (Table 1; Figs. 2, 3). The odds of reoperation were reduced with receipt of NAC in all models except for stage 1 patients with a luminal A subtype. The rates were higher for younger patients with larger tumors and invasive lobular histology. The association between NAC and fewer reoperations was strongest for the patients with the higher breast cancer stage, HER2 overexpression, and triple-negative (TN) cancers. The patients with triple-negative cancers and HER2 overexpression receiving NAC had the lowest ROR (6.4 and 7.3%, respectively). The overall study results did not change in models restricted to patients older than 70 years (data available upon request).

**Table 1:** Association of receipt of neoadjuvant chemotherapy, cancer stage, and breast cancer subtypes with reoperation rates

Patient cohort	Unadjusted ROR			ROR (%)		OR	95% CI		*p* value
	N	D	%	Without NAC	With NAC		Lower	Upper	
Stages 1–3									
Stages 1–3	13444	71627	18.8	20.3	11.4	0.53	0.49	0.57	<0.001
Stage 1	7344	40655	18.1	18.4	13.0	0.65	0.56	0.75	<0.001
Stage 2	5728	28985	19.8	23.1	11.0	0.50	0.45	0.56	<0.001
Stage 3	372	1987	18.7	46.0	11.0	0.27	0.19	0.38	<0.001
Stages 1–3 by type									
Luminal A	5272	22787	23.1	23.6	20.0	0.84	0.76	0.93	0.001
Luminal B1	2701	14066	19.2	20.3	12.8	0.64	0.56	0.74	<0.001
Luminal B2	2413	12416	19.4	21.4	10.6	0.49	0.42	0.57	<0.001
Triple-negative	2177	17617	12.4	14.0	6.4	0.47	0.41	0.55	<0.001
HER2 overexpressed	881	4741	18.6	22.7	7.3	0.34	0.27	0.43	<0.001
Stages 1–3 by type with PSM									
Luminal A	1187	4816	24.6	27.8	21.3	0.74	0.62	0.89	0.002
Luminal B1	662	3170	20.9	25.2	15.5	0.56	0.43	0.73	<0.001
Luminal B2	497	2805	17.7	22.7	13.0	0.49	0.37	0.65	<0.001
Triple-negative	490	4209	11.6	15.6	8.3	0.46	0.36	0.59	<0.001
HER2 overexpressed	211	1112	19.0	19.0	10.7	0.31	0.19	0.50	<0.001
Stage 1 by type									
Luminal A	2828	13891	29.5	20.5	17.8	0.92	0.75	1.12	0.381
Luminal B1	1276	7113	17.9	18.2	13.0	0.73	0.53	0.99	0.044
Luminal B2	1539	7620	20.2	20.7	13.7	0.65	0.50	0.83	<0.001
Triple-negative	1198	9572	12.5	12.9	8.2	0.61	0.46	0.81	<0.001
HER2 overexpressed	503	2459	20.5	21.4	10.2	0.43	0.27	0.70	<0.001
Stage 2 by type									
Luminal A	2307	8453	27.3	28.8	21.1	0.76	0.67	0.87	<0.001
Luminal B1	1323	6509	20.3	22.5	12.4	0.61	0.51	0.73	<0.001
Luminal B2	818	4494	18.2	22.6	9.3	0.46	0.37	0.56	<0.001
Triple-negative	929	7493	12.4	15.6	6.3	0.47	0.39	0.57	<0.001
HER2 overexpressed	351	2036	17.2	25.0	6.6	0.32	0.23	0.44	<0.001
Stage 3 by type									
Luminal A	137	443	30.9	52.0	19.7	0.34	0.21	0.55	<0.001
Luminal B1	102	444	23.0	45.5	14.6	0.37	0.22	0.64	<0.001
Luminal B2	56	302	18.5	51.0	12.0	0.23	0.11	0.49	<0.001
Triple-negative	50	552	9.1	35.7	4.3	0.14	0.07	0.28	<0.001
HER2 overexpressed	27	246	11.0	37.9	7.4	0.35	0.13	0.96	0.041

*ROR* reoperation rates after lumpectomy for invasive breast cancer, *OR* odds ratio, *CI* confidence interval, *N* numerator, *D* denominator; NAC, receipt of neoadjuvant chemotherapy, *HER2* human epidermal growth factor receptor 2, *PSM* propensity score matching

After propensity score matching, each of the matched comparison groups had 8056 patients, including those who received NAC versus those who received chemotherapy after surgery. In these models, the associations between NAC, subtypes and ROR persisted (Table 1; Fig. 3).

In the assessment of tumor response to NAC, the results were unknown for a notable fraction of cases (59% of luminal A, 52% of luminal B1, 49% of luminal B2, 46% of triple-negative, and 42% of HER2 cases). For the cases with known results, the pCR rates after NAC were 17% for the luminal A type, 35.9% for the B1 type, 48.1% for the B2 type, 50.7% for the triple-negative type, and 62.8% for the HER2 type patients. For the patients with a pCR to NAC, the overall ROR for all the subtypes was 4.1%. For the models restricted to the patients not receiving NAC and a separate model including only the patients with a pCR to NAC, the association between subtype and ROR persisted, indicating an intrinsic association between subtype and ROR not dependent on tumor responsiveness to NAC (Table 2; Fig. 3).

**Table 2:** Demonstration of association between breast cancer subtype and reoperation rate independent of the effect of neoadjuvant chemotherapy

	ROR (%)		OR	95% CI		*p* Value	Overall *p* Value
Patient cohort	Subtype	Luminal A		Lower	Upper		
*No NAC (stages 1–3)*							
Triple-negative vs luminal A	14.0	24.0	0.69	0.65	0.73	<0.001	<0.001
HER2 overexpressed vs luminal A	22.7	24.0	1.27	1.16	1.38	<0.001	
Luminal B1 vs luminal A	20.3	24.0	0.94	0.88	0.99	0.028	
Luminal B2 vs luminal A	21.4	24.0	1.11	1.05	1.18	0.001	
*NAC and complete pathologic response (stages 1–3)*							
Triple-negative vs luminal A	2.6	15.5	0.25	0.13	0.47	<0.001	<0.001
HER2 overexpressed vs luminal A	3.1	15.5	0.36	0.17	0.76	0.001	
Luminal B1 vs luminal A	4.2	15.5	0.40	0.19	0.83	0.015	
Luminal B2 vs luminal A	3.8	15.5	0.35	0.18	0.69	0.002	

*ROR* reoperation rates after lumpectomy for invasive cancer, *OR* odds ratio, *CI* confidence interval, *NAC* receipt of neoadjuvant chemotherapy, *HER2* human epidermal growth factor receptor 2

## Discussion

Too many reoperations occur after initial lumpectomy for breast cancer. In the decade ending 2013, nearly one in three women who underwent lumpectomy in the state of New York required a reoperation.^17^ In the United States and Britain, the reoperation rates average 20–25%.^1^–^4^ Rates higher than 50% have been reported, yet some centers report rates lower than 10%, proving that low rates are achievable.^1^–^3^,^18^,^19^ The majority of surgeons participating in a national consensus conference recommended a 10% reoperation rate as a target benchmark.^5^

Our study aimed to assess the association between NAC, breast cancer subtypes, and lumpectomy reoperation rates in a population-based database, including patients who received chemotherapy either before or after surgery, to identify opportunities to lower rates. After adjustment for all covariates known to influence the ROR, we identified a strong association between the receipt of NAC and reductions in reoperations for patients undergoing initial lumpectomy for clinical stages 1–3 breast cancer. Overall, the relative odds of reduction in ROR with chemotherapy before rather than after surgery was 47%. The level of benefit increased with cancer stage. The relative odds of reduction were 35% for stage 1, 50% for stage 2, and 73% for stage 3 patients. The greatest benefit of decreased ROR was seen for patients with the subtypes known to have the highest response rates to chemotherapy (HER2, and TN). For these patients, the relative odds of a reduction in ROR with NAC were respectively 66 and 53% compared to 13 and 36% for the luminal A and B1 types. The unadjusted ROR for the patients with the TN and HER2 subtypes receiving NAC was very low (6.4 and 7.3%, respectively).

Receipt of NAC was associated with fewer reoperations for all age groups, including those patients older than 70 years, a subgroup seldom enrolled in the clinical efficacy trials. The only patient subgroup of any age with no association between NAC and ROR was that including patients who had stage 1 cancer with a luminal A subtype, a cohort not often recommended for chemotherapy according to the National Comprehensive Cancer Network Guidelines except when identified as having a higher risk of cancer recurrence, as measured by the 21-gene multigene signature testing.

Seeking to determine whether the association of cancer subtype with ROR was solely due to its differential rates of response to NAC or whether there could be a distinct independent association of cancer subtype with ROR, we separately calculated ROR by subtype for a cohort of patients who had a pCR to NAC and, using a separate model, for the patients who did not receive any NAC. In both models, an association between subtypes and ROR persisted. This supports the notion that subtypes have an intrinsic association with reoperations, another example of the biologic heterogeneity of breast cancer. Because no reoperations would be expected for positive margins in patients having a complete response to NAC, the few reoperations (4%) that did occur in this cohort were likely due to wound complications, bleeding, or other causes not related to margin status.

Future research into the reason why cancer subtypes may have an independent association with a surgical outcome such as ROR is warranted. Although further investigation is warranted, one reason may be a possible relationship between subtype and tumor focality. If some subtypes are more often multifocal or multicentric, then the likelihood of positive margins necessitating re-excision would be expected to increase. In support of this hypothesis, Pekar et al.,^20^ using large-format histologic techniques, reported that patients with triple-negative tumors had the lowest chance of multifocal or diffuse disease, and those with HER2 subtypes had the highest chance. This result correlates with our findings that for patients not receiving NAC, the triple-negative patients had the lowest ROR (14%), and the HER2 patients had a higher ROR (23%).

The use of NAC is increasing, and evidence shows its effectiveness in increasing breast-conserving therapy rates.^6^ Few studies have reported the effect of NAC on breast reoperations.^21^–^24^ Single-institution reports from Turkey and the University of Michigan associated NAC with a modest reduction in lumpectomy ROR.^22^,^23^ In contrast, Al-Hilli et al.^21^ found no change in ROR for patients undergoing mastectomy captured from the NSQIP database, and Volders et al.^24^ reported higher ROR after NAC for patients undergoing breast-conserving therapy in the Dutch Pathology Registry. The methods of the Dutch study differed from the methods of the current study. In the only prior study of ROR using the NCDB, patients receiving NAC were excluded.^2^

Our study findings are applicable to breast cancer patients either wanting or considering lumpectomy who are otherwise eligible for chemotherapy. Our study was not designed to address the issue of whether a patient should receive chemotherapy. Rather, we aimed to address whether the timing of chemotherapy was associated with reoperation rates in a cohort of patients receiving chemotherapy.

The strengths of this study included the large sample size of the NCDB and its demographic diversity, increasing its generalizability. The concept of discussing chemotherapy sequencing during shared decision making with the patient as a method to lower reoperations should not contribute to the unintended consequences that have been attributed to other initiatives aimed at lowering ROR, such as larger lumpectomies, worse cosmetic outcomes, and increased mastectomy rates from surgeon risk aversion, a fear of being penalized for a reoperation if the surgeon is participating in a quality improvement program.^25^ It could even be postulated that better cosmetic outcomes might result from smaller lumpectomies for patients demonstrating a good clinical response to NAC. For these patients, surgeons do not necessarily need to resect the entire extent of tumor based on its size estimate before NAC, a practice endorsed by the 2015 St. Gallen panel.^9^

This study had limitations. The NCDB is not intended to be a surgical outcomes registry. For example, the NCDB does not have a data field for ROR. It is captured by dates of service for first surgery and definitive surgery. In addition, the NCDB has missing data for some of its fields, including those most notable in this study for assessing tumor response to NAC. These results were missing for about half the cases.

Another limitation was that the NCDB does not record the reported reasons for reoperation described by others.^1^,^3^,^5^,^16^ Our results might differ if any of these confounding variables were unequally distributed between our patients receiving or not receiving NAC. In addition, in this nonrandomized retrospective review, selection bias for the initial decision to counsel a patient to undergo NAC versus surgery and to undergo mastectomy versus lumpectomy after NAC could occur. For example, if the same patient and tumor factors predicting a tumor response to NAC that achieves the goal of breast preservation are similar to those associated with lower ROR, and the patients with poor responses to NAC were sent to the initial mastectomy group, excluding them from our study, then the benefit of NAC lowering ROR in the initial lumpectomy group may be exaggerated. Our secondary analyses attempted to account for the differences in biology (IHC phenotypic subtypes) and patient/tumor characteristics (propensity score matching) that were part of the clinical decision to recommend NAC, but they were insufficient to rule out selection bias.

Finally, in the NCDB and other national databases, some data fields may be misclassified. Investigators cannot determine how often this occurs. It also can be difficult to confirm a cause-and-effect relationship even when strong statistical associations are identified.^6^

## Conclusion

The administration of chemotherapy before instead of after surgery is associated with a highly significant reduction in reoperations after the initial lumpectomy for breast cancer. Benefits are identified with all cancer subtypes but are greatest for patients with cancers classified as TN, HER2, or higher stage disease. A decreased re-excision rate after neoadjuvant chemotherapy represents another advantage to this approach and should be included for an informed discussion of risk and benefits.
